# In Vivo Effects of Joint Movement on Nerve Mechanical Properties Assessed with Shear-Wave Elastography: A Systematic Review and Meta-Analysis

**DOI:** 10.3390/diagnostics14030343

**Published:** 2024-02-05

**Authors:** Gianluca Ciuffreda, Elena Bueno-Gracia, Isabel Albarova-Corral, Alberto Montaner-Cuello, Jorge Pérez-Rey, Pilar Pardos-Aguilella, Miguel Malo-Urriés, Elena Estébanez-de-Miguel

**Affiliations:** 1 Department of Physiatry and Nursing, Faculty of Health Sciences, University of Zaragoza, Calle Domingo Miral S/N, 50009 Zaragoza, Spain; ebueno@unizar.es (E.B.-G.); ialbarova@unizar.es (I.A.-C.); albertomontaner@unizar.es (A.M.-C.); jorge.perez@unizar.es (J.P.-R.); ppardos@unizar.es (P.P.-A.); malom@unizar.es (M.M.-U.); 2 PhysiUZerapy: Health Sciences Research Group, University of Zaragoza, Calle Domingo Miral S/N, 50009 Zaragoza, Spain

**Keywords:** neurodynamics, nerve biomechanics, nerve stiffness, upper limb neurodynamic test, straight leg raise, elastography, shear-wave elastography, ultrasound, ultrasonography

## Abstract

Peripheral nerves are subjected to mechanical tension during limb movements and body postures. Nerve response to tensile stress can be assessed in vivo with shear-wave elastography (SWE). Greater tensile loads can lead to greater stiffness, which can be quantified using SWE. Therefore, this study aimed to conduct a systematic review and meta-analysis to perform an overview of the effect of joint movements on nerve mechanical properties in healthy nerves. The initial search (July 2023) yielded 501 records from six databases (PubMed, Embase, Scopus, Web of Science, Cochrane, and Science Direct). A total of 16 studies were included and assessed with a modified version of the Downs and Black checklist. Our results suggest an overall tendency for stiffness increase according to a pattern of neural tensioning. The main findings from the meta-analysis showed a significant increase in nerve stiffness for the median nerve with wrist extension (SMD [95%CI]: 3.16 [1.20, 5.12]), the ulnar nerve with elbow flexion (SMD [95%CI]: 2.91 [1.88, 3.95]), the sciatic nerve with ankle dorsiflexion (SMD [95%CI]: 1.13 [0.79, 1.47]), and the tibial nerve with both hip flexion (SMD [95%CI]: 2.14 [1.76, 2.51]) and ankle dorsiflexion (SMD [95%CI]: 1.52 [1.02, 2.02]). The effect of joint movement on nerve stiffness also depends on the nerve segment, the amount of movement of the joint mobilized, and the position of other joints comprised in the entirety of the nerve length. However, due to the limited number of studies, many aspects of nerve behavior together with the effect of using different ultrasound equipment or transducers for nerve stiffness evaluation still need to be fully investigated.

## 1. Introduction

The evaluation of the peripheral nervous system normally includes the assessment of conduction and mechanosensitivity [1,2,3,4,5]. In a clinical context, light touch, strength, and reflexes assessment together with electrodiagnostic tests are widely adopted tools to assess neural conduction [1,2,4,5], while a variety of provocative maneuvers like the straight leg raise test or the upper limb neurodynamic test are commonly used for mechanosensitivity assessment [3,6].

Ultrasonography has been increasingly used as an additional examination in patients with suspected peripheral neuropathies [7,8,9,10]. Using conventional ultrasound, one can detect morphological changes in peripheral nerves, quantify their cross-sectional areas, and assess nerve biomechanics [7,8,9,10,11,12]. Despite being a useful technique in the diagnosis of many entrapment neuropathies [7,8,9,10], conventional gray-scale ultrasound is not able to quantify some tissues’ characteristics, such as their mechanical properties.

In the last years, shear-wave elastography (SWE) has been consolidating in the evaluation of tissues’ mechanical properties as an adjunctive technique to conventional ultrasound [13,14,15,16,17,18,19]. SWE is a non-invasive ultrasound imaging technique that uses an acoustic pulse to generate a shear wave in the tissue [13,14,15]. SWE is able to quantify the elastic properties of tissues by assessing the propagation velocity of the shear wave, which is directly related to tissue stiffness [13,14,15,17]. In an elastic, homogeneous, and isotropic medium, the shear modulus (μ) can be estimated with the equation μ = ρc^2^, where ρ is the density (assuming ρ = 1000 kg/m^3^), and c is the speed of the shear wave. Young’s modulus (E) can be approximated as three times the shear modulus: E = 3μ [20]. Consequently, SWE measurements of tissue stiffness are generally reported in m/s as shear-wave velocity (SWV) or converted into kPa as formerly described [13,15,21]. Due to its simplicity and non-invasive character, SWE has already been integrated in many medical disciplines to assess different organs [18,21,22] and musculoskeletal conditions [13,16]. More recently, its application has also been extended to the evaluation of peripheral nerves [16,23,24,25,26,27,28]. The advantage of SWE lies in its capability of recognizing changes in the mechanical properties of tissues related to injury, degeneration, compression, and tension [13,28,29,30,31]. In many neural conditions, the nerve shows an increase in its stiffness in the affected area [23,24,25,26,27,28]. Hence, SWE has been proposed as a novel diagnostic tool for carpal tunnel syndrome [23,25,26], diabetic neuropathy [23,24,25,32,33], and other peripheral neuropathies [23,25,27]. Moreover, SWE may be potentially useful for the prediction of diabetic neuropathy and for the prediction of the risk of diabetic foot ulcers in diabetic neuropathy patients [32,33,34].

However, histopathological changes due to neuropathy are not the single factor affecting nerve stiffness and SWE values [16,35]. A modification of nerve stiffness has also been described in accordance with limb positions aimed to increase or decrease mechanical tension in the nervous system [29,36,37]. Peripheral nerves are exposed to different mechanical loads during limb movements and body postures, and their mechanical response to tensile stress varies depending on nerve location, joint movement, and the position of adjacent joints [11,12,28,38,39]. An increase in nerve stiffness values may be an expression of a greater tensile load applied to a certain neural segment [29,40]. Considering this, SWE may represent a useful tool for the evaluation of in vivo neural response to movement and to define how different patterns impact nerve mechanical properties.

Therefore, this study aimed to conduct a systematic review and meta-analysis to provide an overview of the effect of joint movements on nerve mechanical properties in healthy nerves. We hypothesize that positions and movements that are thought to increase neural tension will lead to higher nerve stiffness values. This information could improve the knowledge of nerve behavior and may allow clinicians to better optimize the mechanical load applied to the neural tissue during both the evaluation and treatment of various neural conditions.

## 2. Materials and Methods

This study was performed in accordance with the Preferred Reporting Items for Systematic Reviews and Meta-Analyses [41]. The protocol was registered in the PROSPERO database with the number CRD42023451960.

### 2.1. Search Strategy

A systematic search was performed in six databases: PubMed, Embase, Scopus, Web of Science, Science Direct, and Cochrane Library. The search was conducted in July 2023 by introducing a combination of keywords and MeSH/Emtree terms related to SWE, peripheral nerves, and joint positioning. The Boolean operators AND and OR were used to combine the search terms. Slight adjustments were made to adapt the search process to each database. The search strategy for each database is detailed in Supplementary Materials.

### 2.2. Eligibility Criteria

This systematic review included studies with primary data of upper/lower extremity nerve stiffness assessed by ultrasound pulse SWE in at least two joint positions in healthy nerves, with a longitudinal or cross-sectional design, published in English, Spanish, or Italian language.

The articles were excluded if they obtained data from other types of elastography (e.g., strain elastography, magnetic resonance elastography, mechanical elastographies, etc.) or only included pathological nerves or nerves with conditions that may affect their biomechanics (e.g., surgery). Studies that did not report original data (reviews, meta-analysis, opinion articles, study protocols, etc.), case reports or case series, letters to the editor, and conference papers were also excluded.

### 2.3. Study Selection

Once the search was performed, duplicates were removed, and potentially relevant articles were identified from the titles and abstracts. The eligibility was determined by reading the full text and considering the inclusion and exclusion criteria proposed for this systematic review.

The systematic search, study selection, and data extraction were performed by two independent researchers (G.C. and I.A.-C.). Any doubts or disagreements were resolved by a third researcher (E.E.-d.-M.).

For each included study, the following data were extracted: author, year of publication, characteristics of the sample, number of subjects, nerve, point of measure, ultrasound system, transducer orientation, region of interest (ROI), joint position, and results obtained. If a study presented relevant data only in graphic format, the information was extracted from figures with the online tool WebPlotDigitizer [42]. In case clarification was needed, the authors of the study were contacted.

### 2.4. Quality of the Studies

Two researchers (G.C. and I.A.-C.) independently assessed the quality of the included studies. The assessment was performed with a modified version of the Downs and Black checklist [43]. This tool was previously used in systematic reviews of in vivo biomechanical properties of peripheral nerves [11,12]. Individual items were either rated as “yes” (=1) when properly described or “no/unable to determine” (=0) if not addressed in the study or if the raters could not determine it. The total quality score was reached by final consensus, with a maximum score of 17. A total score of ≤7 was considered as low quality, 8–11 as fair quality, and >11 as good quality. This modified scoring system was originally described by Fernando et al. [44] and successively adopted in other systematic reviews [11,12].

### 2.5. Meta-Analysis

We performed the meta-analysis when a minimum of two studies were considered comparable. Studies were considered comparable when they reported SWE values (in m/s or kPa) for similar nerve locations and similar joint movement. Standardized mean difference (SMD) was used as a measure of effect size. If standard error (SE) was reported instead of standard deviation (SD), SD was obtained using the following formula: SD=SE×√N. In case a study presented the mean and its 95% confidence interval (CI), the respective SD was calculated by dividing the width of the CI by 3.92 and then multiplying by the square root of the sample size in that group; for small sample sizes, 3.92 was substituted with a t distribution value [45]. If median, interquartile range, minimum, and maximum values were reported, mean and SD were estimated using the methods developed by Luo et al. [46] and Shi et al. [47] after checking for the absence of significant data skewness [48]. If significant skewness was detected, the data were not included in the meta-analysis [48]. When a study presented data separately for subgroups (e.g., right and left nerves, young and old participants, etc.) the following formulae were used to combine numbers into a single sample size, mean, and SD [45]:

$$Sample\;size=N_{1}+N_{2};\;Mean=\frac{N_{1}M_{1}+N_{2}M_{2}}{N_{1}+N_{2}};$$

$$SD=\sqrt{\frac{(N_{1}-1)\;SD_{1}^{2}+(N_{2}-1)\;SD_{2}^{2}+\frac{N_{1}N_{2}}{N_{1}+N_{2}}\;\left(M_{1}^{2}+M_{2}^{2}-2M_{1}M_{2}\right)}{N_{1}+N_{2}-1}}$$

Separate analyses were performed depending on nerve location and joint positions. In case a study reported nerve SWE values at multiple points of measure of the same nerve segment, the most similar location to the one adopted in other included studies was used for the analysis. Baseline data were analyzed in studies with a longitudinal design.

Two studies performed by the same group shared part of the sample, where the same healthy participants who represented the entirety of the sample in one study were used as control group in a second one [49,50]. To avoid introducing duplicated participants in the meta-analysis, we extracted data from healthy subjects in one study [50], and in the other, we analyzed SWV values of the unaffected leg in the patients’ group [49].

Considering the expected heterogeneity between studies, random effect models were applied, weighing each effect size by the inverse of its variance, with this being defined as the sum of the intra-study and inter-study variances. The statistical analysis comprised the calculation of the mean effect size with its 95% confidence interval as well as the calculation of the *I*^2^ to evaluate the level of heterogeneity among studies. Forest plots were constructed to represent the results. To check the robustness of the findings, sensitivity analysis was performed by removing one study at a time from each comparison with at least 3 studies. Due to the low number of studies included in each comparison, the investigation of quantitative or qualitative moderators through sub-group analysis and meta-regression was not performed, and publication bias was not assessed [45,51]. If meta-analysis for a specific comparison was not possible due to insufficient data, the results of the studies were narratively described. All meta-analyses were conducted using RevMan 5.4 software.

## 3. Results

### 3.1. Study Selection

**Figure 1:**
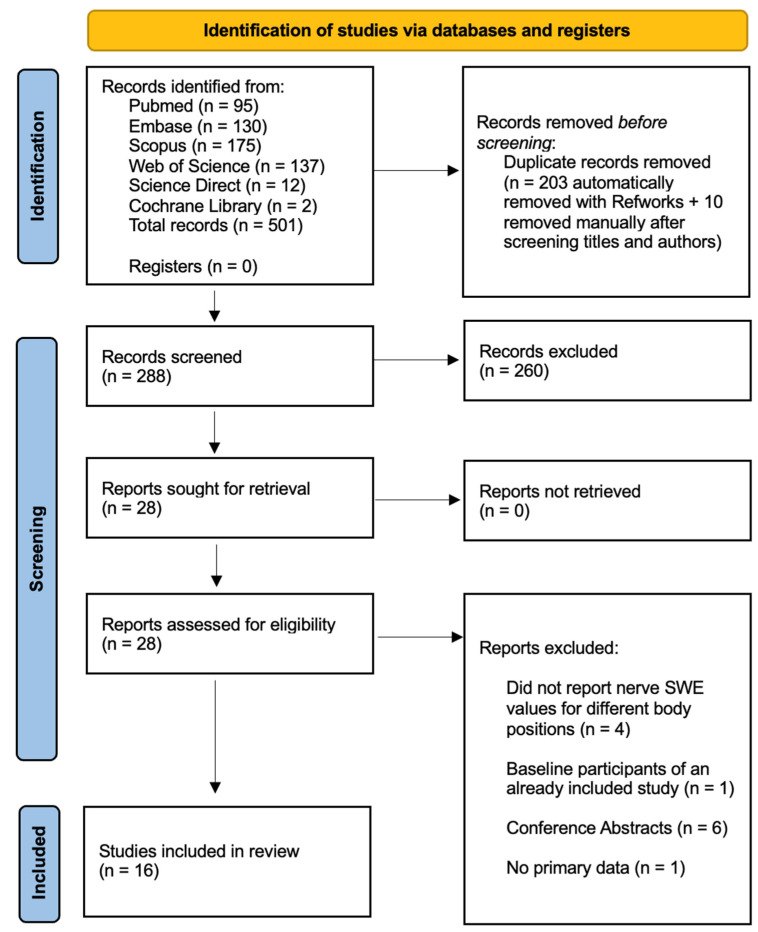
PRISMA 2020 flow chart illustrating the study selection process.

A total of 501 records were obtained from our initial search. After the removal of duplicates, 288 studies remained. Following the screening of titles and abstracts, 260 studies were excluded. After reading 28 full-text papers, 12 were discarded for not meeting the inclusion or exclusion criteria. Finally, 16 studies were included in the current systematic review (Figure 1).

### 3.2. Quality of the Studies

The methodological quality of the included studies is summarized in Table 1. The modified Downs and Black checklist score varied from 9 to 13. Five studies showed good quality (more than 11 points) [49,52,53,54,55], and 11 showed fair quality (8–11 points) [36,37,50,56,57,58,59,60,61,62,63]. None of the included studies scored below 9 points. The included studies mostly lacked reporting subjects’ representativeness, blinding of the assessors, and sample size calculation.

### 3.3. Characteristics of the Studies

The characteristics of the 16 included studies are shown in Table 2 and Table 3.

In the upper extremity, the median nerve (MN) was assessed in six studies [36,37,55,61,62,63], while the ulnar nerve (UN) was explored in three studies [37,57,60]. In the lower extremity, the sciatic nerve (SN) and the tibial nerve (TN) were evaluated in six studies [49,50,53,54,58,59] and four studies [36,52,54,56], respectively. The MN was most commonly imaged in the forearm [36,37,55,61,62], followed by the proximal elbow [36,37], and lastly at the carpal tunnel level [63]. The UN was evaluated in the elbow region in three studies [37,57,60], and one study also reported data at the forearm level [37]. The SN was assessed in the proximal thigh in all six studies [49,50,53,54,58,59], and one study also assessed it at the mid-thigh level [54]. The TN was imaged in four studies at the distal portion of the leg [36,52,54,56], and among them, one study also assessed it in the popliteal fossa and in the proximal part of the leg [54].

The most used ultrasound system was the Aixplorer Supersonic Imagine, which was employed in 10 studies [37,49,50,54,55,57,58,59,60,62]; the remaining studies used the Canon Aplio [52,56,61] and the Siemens Acuson [36,53,63]. All ultrasound systems were coupled with a lineal probe [36,37,49,50,52,53,54,55,56,57,58,59,60,61,62,63].

Fourteen studies performed nerve measurements in long axis [36,37,49,50,52,53,54,55,56,58,59,60,61,62], and two performed them in short axis [57,63]. Different regions of interest (ROIs) were used for nerve SWE assessment. In seven studies, the largest nerve area possible was used as the ROI [37,49,50,53,54,58,59]; five studies performed quantitative SWE measurements with a 2 mm ROI [55,60,61,62,63]; and three studies used multiple ROIs. Among them, two used three randomly selected ROIs [52,56], and one used four 1.5 mm ROIs along the nerve [36]; one study did not specify the characteristics of the ROI used [57]. Thirteen of the included studies reported SWE values in m/s [36,37,49,50,52,53,54,55,56,58,59,61,63], while three expressed SWE values in kPa [57,60,62].

One study [64] was excluded because its baseline was analyzed in an already included study [54].

### 3.4. Median Nerve

Results from the studies that analyzed the effects of joint movement/positioning on MN stiffness are summarized in Table 4.

#### 3.4.1. Effect of Wrist Movement

**Figure 2:**
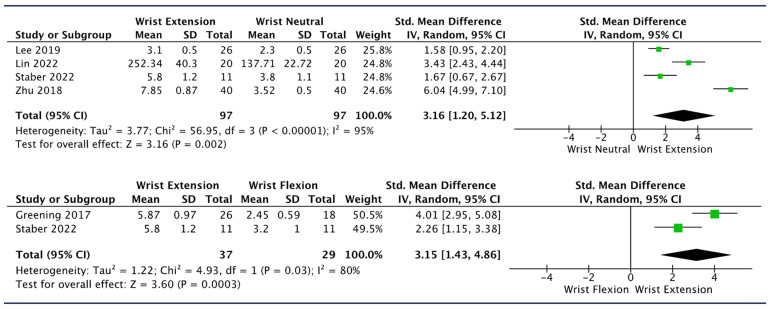
Forest plots showing the impact of different wrist positions on SWE values of distal median nerve (forearm and wrist) [36,55,61,62,63].

The effect of wrist extension on MN SWE values was analyzed in five studies [36,55,61,62,63]. Meta-analysis showed that wrist extension significantly increased MN stiffness in its distal segment (wrist and forearm) regardless of starting from wrist flexion or wrist neutral position, with similar pooled effect sizes (SMD [95%CI]: 3.15 [1.43, 4.86], I^2^: 80%; SMD [95%CI]: 3.16 [1.20, 5.12], I^2^: 95%) (Figure 2).

Sensitivity analysis revealed that by removing the study of Zhu et al. [55] from the “wrist extension vs. wrist flexion” comparison, the pooled effect size decreased by 31%, and the heterogeneity decreased by 15%; however, it remained strongly in favor for wrist extension (SMD [95%CI]: 2.19 [1.07, 3.31], I^2^: 80%) (Table 5).

Lin et al. [62] reported the highest MN stiffness values at the forearm level when wrist extension was combined with cervical contralateral lateral flexion starting from a shoulder abduction and elbow extension position (starting position: 137.71 ± 22.72 kPa, only wrist extension: 252.34 ± 40.30 kPa, only contralateral cervical flexion: 211.00 ± 30.49 kPa, wrist extension and contralateral cervical flexion: 297.35 ± 64.60 kPa; *p* < 0.001 for all movements vs. starting position). Lee et al. [63] reported that wrist extension led to higher nerve SWV values at carpal tunnel when compared to wrist at 0° regardless of the fingers’ position. However, the difference was significant only in respect to wrist at 0° with the fingers in neutral position (*p* < 0.001).

Additionally, one study that analyzed the effect of wrist movement on MN stiffness in the proximal elbow and in the forearm reported changes in SWV at both locations when adding wrist extension in a position of shoulder abduction and elbow flexion; however, the greatest increase in SWV was observed at the forearm level [36].

#### 3.4.2. Effect of Finger Movement

One study assessed the effect of different finger positions on MN SWV at carpal tunnel level [55]. With the wrist in neutral position, both finger grasp and finger extension led to an increase in nerve SWV with respect to the neutral position (mean SWV ± SD (m/s): finger neutral 2.3 ± 0.5; finger grasp 2.7 ± 0.5; finger extension 2.7 ± 0.4; *p* < 0.05); during wrist extension, higher SWV values were observed for finger extension with respect to finger grasp and neutral positions, although the difference among these positions was not significant (mean SWV ± SD (m/s): finger neutral 2.9 ± 0.5; finger grasp 3.0 ± 0.5; finger extension 3.1 ± 0.4) [55].

#### 3.4.3. Effect of Elbow Movement

Two studies investigated the effect of elbow movement on MN SWV in the upper arm and in the forearm, showing that performing an elbow extension from a 90° flexion angle led to a substantial increase in SWV in both locations [36,37]. Meta-analysis was not performed because data from one study [37] ended up being significantly skewed. Therefore, the SD from the median, interquartile range, and minimum and maximum values graphically reported in the study was not estimated in the present study [48].

#### 3.4.4. Effect of Shoulder Movement

The impact of shoulder movement was investigated in one study, which reported that abducting the shoulder with the elbow and wrist flexed caused negligible changes in MN SWV in the upper arm and forearm [36].

#### 3.4.5. Effect of Cervical Movement

One study assessed MN stiffness in response to cervical contralateral lateral flexion [62]. At the initial position of 90° of shoulder abduction and elbow extension, adding cervical contralateral lateral flexion increased nerve stiffness in the forearm from 137.71 ± 22.72 kPa to 211.00 ± 30.49 kPa with the wrist in neutral position and from 252.34 ± 40.30 kPa to 297.35 ± 64.60 with the wrist extended [62].

### 3.5. Ulnar Nerve

The results of the studies that investigated the UN are summarized in Table 6.

#### Effect of Elbow Flexion

**Figure 3:**
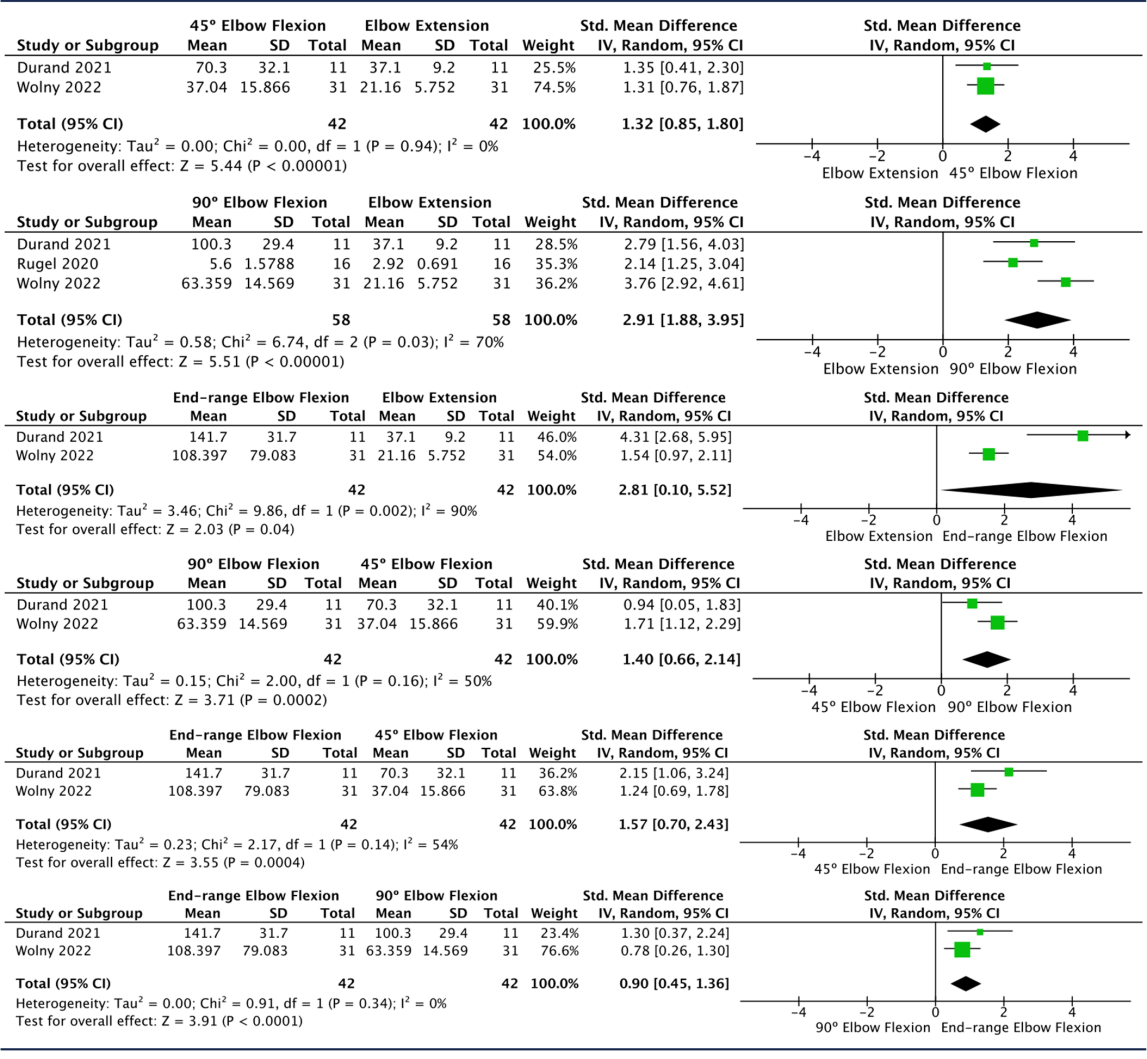
Forest plots showing the impact of different elbow positions on ulnar nerve SWE values at the elbow location [37,57,60].

Three studies evaluated UN stiffness at different degrees of elbow flexion [37,57,60]. Meta-analysis showed a trend of progressive increase in UN stiffness in the elbow region with greater flexion angles. The largest effect sizes were observed comparing both 90° elbow flexion and end-range elbow flexion to elbow extension (SMD [95%CI]: 2.91 [1.88, 3.95], I^2^: 70%; SMD [95%CI]: 2.81 [0.10, 5.52], I^2^: 90%) (Figure 3). Sensitivity analysis was performed only for the “90° elbow flexion vs. elbow extension comparison.” After removing the study by Rugel et al. [37] from the analysis, the effect size was 16% higher and the heterogeneity decreased (SMD [95%CI]: 3.39 [2.46, 4.31], I^2^: 38%); after removing Wolny et al. [57], the pooled effect size decreased by 19% with no heterogeneity (SMD [95%CI]: 2.37 [1.64, 3.09], I^2^: 0%) (Table 5).

Additionally, one study observed a similar behavior of the UN at the forearm level, reporting an increase in SWV values with 90° elbow flexion compared to elbow extension [37].

### 3.6. Sciatic Nerve

SN stiffness behavior in response to joint motion is summarized in Table 7.

#### 3.6.1. Effect of Ankle Movement

Five studies measured SN SWV at different ankle angles with knee extension [49,50,53,58,59]. As these studies used different methods to express ankle angles (see Table 7), the SWV values reported at each given angle were normalized to a common scale of 0 to 100% of dorsiflexion, where 0% represented 40° of plantar flexion and 100% represented the maximum dorsiflexion. The data available for the most similar %-of-dorsiflexion angle among the mentioned studies were used to meta-analyze SN SWV in ankle dorsiflexion and ankle plantar flexion. In the comparisons of ankle dorsiflexion/plantar flexion vs. ankle in neutral position (≈0°), one study was not included due to the impossibility of estimating which percentage of the reported dorsiflexion angle corresponded to a position ≈ 0° [59].

**Figure 4:**
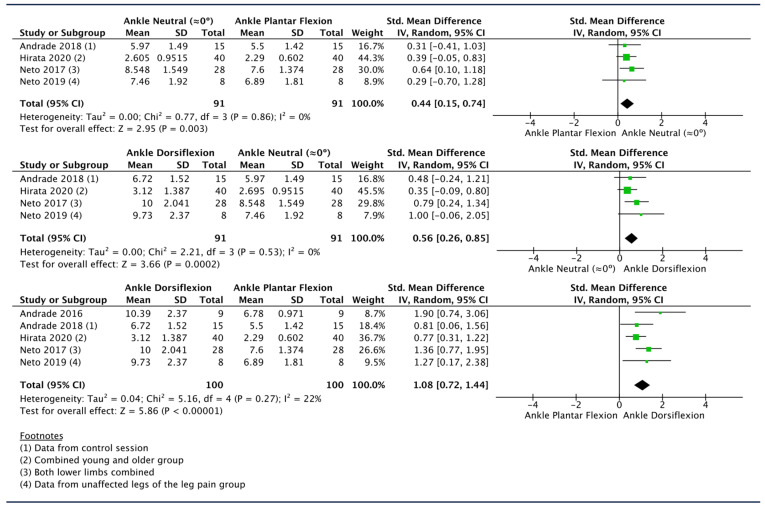
Forest plots showing the impact of different ankle positions on sciatic nerve SWE values in the thigh [49,50,53,58,59].

The pooled SMDs obtained from the comparisons of ankle plantar flexion vs. neutral position and neutral position vs. ankle dorsiflexion showed a similar moderate effect size, suggesting a progressive increase in SN SWV as the ankle angle becomes greater (SMD [95%CI]: 0.44 [0.15, 0.74], I^2^: 0%; SMD [95%CI]: 0.56 [0.26, 0.85], I^2^: 0%). As expected, the greatest change in SWV was found comparing ankle plantar flexion vs. ankle dorsiflexion (SMD [95%CI]: 1.08 [0.72, 1.44], I^2^: 22%) (Figure 4).

Sensitivity analysis revealed that the largest modification of the effect size is obtained by removing the study by Hirata et al. [53] from the dorsiflexion vs. neutral and plantar flexion comparisons and the study by Neto et al. [50] from the plantar flexion vs. neutral comparison, which led to changes in the mean effect size by 30%, 17%, and 18%, respectively (Table 5).

However, the impact of ankle movement on SN stiffness may vary depending on knee position. As one study observed, at the 90° knee flexion position, ankle dorsiflexion seemed not to produce any change in SN SWV in the thigh [59].

#### 3.6.2. Effect of Hip Movement

One study analyzed the impact of hip movement on SN stiffness at proximal and mid-thigh levels, showing that hip flexion led to a uniform average increase in SWV values by 54% from the starting position in hip extension (*p* < 0.0001) [54].

#### 3.6.3. Effect of Knee Movement

SN SWV in response to progressive ankle dorsiflexion at 90° knee flexion and with full knee extension was investigated in one study [59]. According to the results of this study, SN SWV showed significantly greater values in knee extension vs. knee flexion regardless of the ankle angle (*p* values ranging from 0.001 to 0.002) [59].

### 3.7. Tibial Nerve

The studies analyzing the impact of different joint movements on TN SWE values are summarized in Table 8.

#### 3.7.1. Effect of Ankle Movement

**Figure 5:**
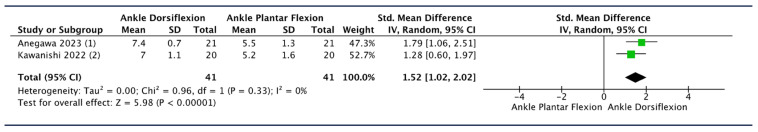
Forest plot showing the impact of different ankle positions on tibial nerve SWE values in the lower leg [52,56].

Two studies evaluated TN stiffness in response to isolated ankle movement in a 30° knee flexion position [52,56], while one study analyzed a combination of ankle dorsiflexion and knee extension [36]. Meta-analysis was performed with the data of the first two studies. Mean effect size showed a large increase in TN SWV with ankle dorsiflexion movement when compared to ankle resting position in plantar flexion (SMD [95%CI]: 1.52 [1.02, 2.02], I^2^: 0%) (Figure 5). A similar effect was observed when performing a combination of ankle dorsiflexion and knee extension (mean SWV ± SEM: 5.16 ± 0.21 m/s) from a starting position with 90° knee flexion and foot relaxed (mean SWV ± SEM: 3.25 ± 0.10 m/s) [36].

#### 3.7.2. Effect of Hip Movement

**Figure 6:**
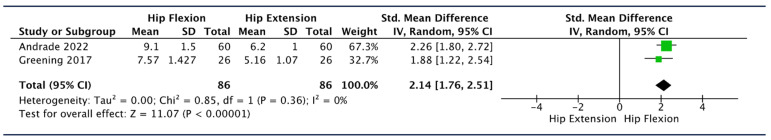
Forest plots showing the impact of different hip positions on tibial nerve SWE values in the lower leg [36,54].

Two studies assessed the effect of hip flexion movement on TN SWV [36,54]. In both studies, the ankle was in a dorsiflexion position with the knee extended [36,54]. Pooled SMD revealed that hip flexion led to greater SWV values compared to hip extension in the distal portion of the TN (SMD [95%CI]: 2.14 [1.76, 2.51], I^2^: 0%) (Figure 6). Similar behavior was also observed in more proximal locations of the TN [54].

#### 3.7.3. Effect of Knee Movement

The impact of knee movement on TN stiffness was reported in one study, which observed a 60% increase in TN SWV when performing knee extension in combination with ankle dorsiflexion starting from 90° knee flexion, with foot relaxed and hip neutral [36].

## 4. Discussion

The current systematic review and meta-analysis aimed to investigate the effects of joint movement on stiffness in healthy nerves. We hypothesized that nerve stiffness would significantly increase with movements and positions associated with more tensile load on neural structures. The results of this systematic review and meta-analysis support our initial hypothesis showing an overall tendency of stiffness increase following a pattern of neural tensioning. The effect of joint movement on nerve stiffness depends on the nerve segment, the amount of movement of the joint mobilized, and the position of other joints comprised in the entirety of the nerve length. These results are in line with previous systematic reviews that assessed nerve biomechanics [11,12].

The impact of neural-tensioning movement on nerve stiffness seems to be dependent on the amount of motion assumed by the joints involved. However, the relationship between joint angle and the increase in nerve stiffness may not always be completely linear through the entire range of motion. Some nerve segments show a gradual increase of stiffness as the joint is progressively moved [57,60], while others exhibit a different pattern, with an increase starting from a certain joint angle or that depends considerably on adjacent joint positioning [49,50,52,59,61].

Individual studies reported that a progressive increase in elbow flexion angles is associated with higher UN stiffness at the elbow [57,60]. In line with this, meta-analysis reveals significant effect sizes for higher degrees of elbow flexion when comparing 0° vs. 45°, 45° vs. 90°, and 90° vs. full flexion, suggesting that gradually increasing the elbow flexion angle could imply a further increase in UN stiffness (SMD [95%CI]: 1.32 [0.85, 1.80], I^2^: 0%; 1.40 [0.66, 2.14], I^2^: 50%; 0.90 [0.45, 1.36], I^2^: 0%). Surprisingly, in the analysis of elbow extension with other elbow flexion angles, the highest pooled SMD was found in the 0° vs. 90° comparison rather than in the 0° vs. full flexion comparison (SMD [95%CI]: 2.91 [1.88, 3.95], I^2^: 70%; SMD [95%CI]: 2.81 [0.10, 5.52], I^2^: 90%), as one could initially expect based on the previous findings. However, it must be considered that the width of the pooled 95% confidence interval for the SMD of the 0° vs. full flexion comparison may indicate imprecision of the estimated pooled effect size.

Wrist extension produced a significant increase in nerve stiffness in the distal segment of the MN [36,55,61,62,63] (Figure 2). Despite the presence of heterogeneity, all studies included in the analysis showed a large effect size in favor of wrist extension. Sensitivity analysis indicated that by removing the study by Zhu et al. [55], the pooled effect size reduced by 31%; nevertheless, this did not substantially change the clinical implication of the results obtained (Table 5). These findings are consistent with the increase in MN strain observed when performing similar joint movements in both cadaveric and in vivo studies [65,66]. Interestingly, the meta-analysis revealed a similar pooled effect size when comparing wrist extension to both neutral position (0°) and wrist flexion. This could indirectly suggest that the range from wrist flexion to neutral position does not seem to significantly affect nerve stiffness. Although a meta-analysis for a direct comparison was not performed due to insufficient data, the results from one study reporting a non-significant difference between these two wrist positions may support these assumptions [61]. In line with this, Silva et al. suggested that wrist movements from flexion to 0° induced less MN gliding than movements from 0° to wrist extension [11]. One possible explanation for this is that the range from wrist flexion to neutral position did not imply a sufficient level of mechanical stress to change MN stiffness, i.e., the nerve was unloaded [65]. However, care must be taken to interpret these suggestions, because wrist flexion vs. neutral was not directly meta-analyzed, and wrist flexion vs. extension was only reported in studies where participants’ elbows stayed in a flexed position (MN unloaded), and results may substantially differ if the same movements were performed in other limb positions implying more neural tension.

Regarding the SN, the meta-analysis revealed a progressive increase in stiffness at the thigh level with ankle dorsiflexion movement in a position of knee extension. Significant pooled SMD values were observed for ankle dorsiflexion and ankle neutral position (≈0°) when compared to ankle plantar flexion (SMD [95%CI]: 1.08 [0.72, 1.44], I^2^: 22%; SMD [95%CI]: 0.44 [0.15, 0.74], I^2^: 0%) as well as for ankle dorsiflexion compared to neutral position (SMD [95%CI]: 0.56 [0.26, 0.85], I^2^: 0%) (Figure 4). As expected, the ankle dorsiflexion vs. ankle plantar flexion comparison presented a larger effect size. These results seem to indicate a linear increase in SN stiffness related to the amount of ankle dorsiflexion movement. However, several included studies also reported non-significant changes in SN in the range from 40° ankle plantar flexion to 40% of maximum ankle dorsiflexion, being 50% of ankle dorsiflexion, the starting point from which the SN starts to build up more tension, while lower ankle dorsiflexion angles are likely to keep the nerve unloaded [49,50,59]. In the present meta-analysis, the extracted mean (± SD) SWE values for ankle neutral position were ≥50% of subjects’ maximum ankle dorsiflexion, consequently the first phase of ankle dorsiflexion movement, when the nerve is unloaded, was obviated in the analysis and thus not reported quantitatively in this review.

The TN showed a similar pattern, with nerve SWV increasing with ankle dorsiflexion (SMD [95%CI]: 1.52 [1.02, 2.02], I^2^: 0%) (Figure 5). However, similarly to the MN [61] and SN [49,50,59], the TN exhibited an initial phase of unloading during ankle movement. One study found that the TN SWV increase was only significant beyond a certain point of the plantar flexion-dorsiflexion range of motion (75% of maximum dorsiflexion), as the initial phase of the range of motion did not significantly affect nerve stiffness [52]. As the TN is located closer to the mobilized than the SN, the threshold from which the TN starts building tension (75%) may be expected in a smaller dorsiflexion angle position compared to the SN (50%). This may be due to the different limb positioning. The TN was measured with 30° knee flexion and 90° hip flexion [52], while the SN was measured with knee and hip extension [49,50,59]. The angle of knee and hip flexion may have supposed less tensile load on the TN than the angle of knee and hip extension did for the SN; therefore, if this is the case, more ankle dorsiflexion might be required to produce the same neural tension. A study on cadavers investigated the effect on TN strain of ankle dorsiflexion in different combinations of hip and knee positions and reported no significant differences in the final position when performing ankle dorsiflexion in a hip flexion-knee flexion position or a hip extension-knee extension position; however, the initial strain was different between the two positions, which may indicate that TN may experiment with a different pattern of tensile load depending on limb positioning (initial strain: −3.6% for hip flexion-knee flexion; −1.3% for hip extension-knee extension; expressed as % of change from the reference position) [67]. Regardless, further investigations are needed to fully understand cumulative nerve tensioning produced by the position of the joints that it crosses.

Limb positioning and its effect on neural tension and stiffness may also vary depending on the nerve segment considered.

MN seems to exhibit a greater increase in SWV in the proximal elbow (89.3%) than in the forearm (64.0%) during elbow extension [37], while wrist extension is more likely to produce a larger increase in SWV in the forearm (127%) than in the proximal elbow (40%) [36]. A similar pattern of change was observed for the UN, with a 91.1% increase in SWV at the proximal elbow and a 37.4% increase at the forearm when the elbow was flexed [37]. These findings for the MN and UN may indicate that changing the position of the limb could lead to changes in stiffness that vary along the nerve path. The large difference in stiffness increase between the forearm and the upper arm in these positions could reflect more tension in the nerve segment closer to the moving joint.

On the other hand, one study reported the effect of hip flexion on different portions of the sciatic/tibial nerve and observed a homogeneous average 54% increase in SWV along the SN and TN [54]. However, it must be considered that in this study, SN and TN were not fully unloaded due to the knee extension and ankle (0°) angles, so a partial pre-tensioned status of the nerve could have influenced its pattern of stiffness modification during hip flexion. In line with this, a previous cadaveric study reported a greater increase in TN strain at the ankle during the first phase of the straight leg raise test, when the first movement was ankle dorsiflexion compared to hip flexion [68].

The main strength of this systematic review lies in its synthesis of in vivo nerve biomechanics from both qualitative and quantitative perspectives. The direction of the effect is consistent among the included studies, showing an increase in nerve stiffness in response to tension-increasing movements. The exclusive inclusion of studies that used SWE, which is considered less operator-dependent than other types of elastographies such as strain elastography [30], may have contributed to the consistency of the observed effect and represent another point of strength.

Beside this, several limitations need to be considered. First, the limited number of studies included in the meta-analyses did not allow a proper investigation of moderator variables such as age, the position of adjacent joints, the ultrasound system used, or the transducer position; thus, the heterogeneity reported for various comparisons could not be fully explained. Despite the presence of heterogeneity for some comparisons, sensitivity analysis indicated robustness of the results, showing that no individual study had a significant impact on the entire analysis or produced substantial changes that might affect the clinical interpretation of the results. Furthermore, the methodological quality of the included studies was assessed with a modified version of the Downs and Black checklist. Although this scale was adopted in previous similar reviews [11,12], its clinometric properties have not yet been studied. Nevertheless, it was adopted because other quality assessment tools seem to be less adequate than this modified checklist.

Another relevant aspect to take into account is that under mechanical tension, nerve SWV exhibits higher values, which is assumed to imply an increase in its stiffness [29]. However, the actual amount of neural tension cannot be non-invasively quantified nor isolated from the effects of other forces acting upon the nerve, and thus resultant nerve stiffness may also be influenced by interactions of the nerve with its surrounding tissues. Lastly, this systematic review focused exclusively on describing and analyzing healthy nerve behavior. Nerves under pathological conditions may present different mechanical properties in response to joint movement [57,61]; therefore, our findings must be interpreted in this context and cannot be generalized to pathological nerves. Future systematic reviews may investigate how different types of movements may affect nerve stiffness in different pathologies.

## 5. Conclusions

Peripheral nerves are exposed to different mechanical loads during limb movements, and their mechanical response to tensile stress is variable. Shear-wave elastography may represent a useful tool for the evaluation of in vivo neural response to movement and to define how different patterns impact nerve mechanical properties. Taking this into account, the present systematic review and meta-analysis aimed to investigate the effects of joint movement on nerve stiffness.

Our findings suggest an overall tendency for stiffness increase following a pattern of neural tensioning, where greater stiffness values were observed for movement and positions associated with more tensile load on neural structures. The effect of joint movement on nerve stiffness is complex and depends on the nerve segment, the amount of movement of the joint mobilized, and the position of other joints comprised in the entirety of the nerve length. However, due to the limited number of studies, many aspects of the nerve behavior, such as the cumulative effect produced by the position of many joints, together with the effect of using different ultrasound equipment and transducers for nerve stiffness evaluation, still need to be fully investigated.

## Figures and Tables

**Table 1:** Modified Downs and Black checklist for quality assessment.

	Lee 2019 [63]	Lin 2022 [62]	Staber 2022 [61]	Zhu 2018 [55]	Greening 2017 [36]	Rugel 2020 [37]	Durand 2021 [60]	Wolny 2022 [57]	Andrade 2016 [59]	Andrade 2018 [58]	Andrade 2022 [54]	Hirata 2020 [53]	Neto 2017 [50]	Neto 2019 [49]	Kawanishi 2022 [52]	Anegawa 2023 [56]
1. Hypothesis/aim/objective	1	1	0	1	1	1	1	1	1	1	1	1	1	1	1	1
2. Main outcomes	1	1	1	1	1	1	1	1	1	1	1	1	1	1	1	1
3. Participants’ characteristics	1	1	01	1	1	1	1	1	1	1	1	1	01	1	1	1
5. Confounders	0	1	1	1	1	1	0	0	1	1	1	1	1	1	0	0
6. Findings	1	1	1	1	1	1	1	1	1	1	1	1	1	1	1	1
7. Estimates random variability	1	1	1	1	1	1	1	1	1	1	1	1	1	1	1	1
10. Actual probability values	1	1	1	1	0	0	0	1	1	1	1	1	1	1	1	1
11. Subjects’ representative (asked)	0	0	0	0	0	0	0	0	0	0	0	0	0	0	0	0
12. Subjects’ representative (agreed)	0	0	0	0	0	0	0	0	0	0	0	0	0	0	0	0
15. Blinding of assessors	01	01	0	0	0	0	0	1	0	0	0	0	0	0	0	0
16. Data dredging	1	1	1	1	1	1	1	1	1	1	1	1	1	1	1	1
18. Appropriate statistical tests	1	1	1	1	1	1	1	1	1	1	1	1	1	1	1	1
20. Outcome measures valid/reliable	1	1	1	1	1	1	1	1	1	1	1	1	1	1	1	1
21. Internal validity (selection bias)	01	01	0	0	0	1	1	1	0	0	1	0	0	0	1	0
22. Recruitment time period	1	0	0	1	0	0	1	0	0	0	1	0	0	0	1	0
25. Adjustment for confounding	0	1	1	1	1	1	0	0	1	1	1	1	1	1	0	0
27. Statistical power determined	1	0	0	0	0	0	0	0	0	0	0	1	0	1	1	0
Total G.C. vs. Total I.A.-C.	11 vs. 13	11 vs. 13	9 vs. 10	12 vs. 12	11 vs. 11	11 vs. 11	10 vs. 10	11 vs. 11	11 vs. 11	11 vs. 11	13 vs. 13	12 vs. 12	10 vs. 11	12 vs. 12	12 vs. 12	9 vs. 9
Total consensus	11	11	9	12	11	11	10	11	11	11	13	12	10	12	12	9

1 = considered appropriately addressed by both assessors; 0 = considered inappropriately addressed by both assessors; 10 = considered appropriately addressed by only the first assessor; 01 = considered appropriately addressed by only the second assessor.

**Table 2:** Characteristics of the included studies.

	Author and Year	N	Sample Characteristics	Age (Years)	Gender (M/F)
***Median nerve***					
	Zhu 2018 [55]	40	Healthy subjects	31.20 ± 8.92	13/27
	Greening 2017 [36]	26 (N = 18 for position 2 of MN evaluation)	Healthy subjects	Men 37.5 (20–72); Women 38.8 (23–58).	11/15
	Lin 2022 [62]	20	Healthy subjects	19.9 ± 1.4	7/13
	Lee 2019 [63]	26	Healthy subjects	24.7 ± 3.7	20/6
	Staber 2022 [61]	11	Healthy controls	30.6 ± 11.0	-
	Rugel 2020 [37]	16	Healthy subjects	24.9 ± 2.2	10/6
***Ulnar nerve***					
	Wolny 2022 [57]	31	Healthy contralateral nerves from patients with unilateral ulnar tunnel syndrome	54.2 ± 8.15	-
	Durand 2021 [60]	11	Nerves from the contralateral healthy side, in patients with UN decompression with anterior transposition	53.2 ± 14.9	6/5
	Rugel 2020 [37]	16	Healthy subjects	24.9 ± 2.2	10/6
***Sciatic nerve***					
	Hirata 2020 [53]	20 20	Young males Older males	22 ± 1 (young) 72 ± 5 (old)	40/0
	Neto 2019 [49]	8	Patients with chronic unilateral low back related leg pain	30.8 ± 7.4 (leg pain)	6/2 (leg pain)
		8	Healthy controls	28.1 ± 8.3 (healthy controls)	5/3 (healthy controls)
	Neto 2017 [50]	14	Healthy subjects	30.4 ± 10.1	11/3
	Andrade 2016 [59]	10	Healthy subjects	25.3 ± 2.5	10/0
	Andrade 2022 [54]	60	Healthy subjects	20.5 ± 2.0	29/31
	Andrade 2018 [58]	15	Healthy subjects	22 ± 3	13/2
***Tibial nerve***					
	Kawanishi 2022 [52]	20	Healthy subjects	23.8 ± 5.5	14/6
	Anegawa 2023 [56]	21	Healthy subjects	20.8 ± 0.5	10/11
	Greening 2017 [36]	26	Healthy subjects	Men 37.5 (20–72); Women 38.8 (23–58).	11/15
	Andrade 2022 [54]	60	Healthy subjects	20.5 ± 2.0	29/31

M = males; F = females; MN = median nerve.

**Table 3:** Methodological characteristics of the included studies.

	Author and Year	Ultrasound Machine	Probe	Plane	Point of Measure	Measurement Methods	Unit
***Median nerve***							
	Zhu 2018 [55]	Aixplorer	4 to 15-MHz linear array probe	L	Mid-forearm	Circular ROI of 2 mm	m/s
	Greening 2017 [36]	Siemens Acuson S2000	4–9 MHz 38.5 mm linear array transducer	L	(1) mid-forearm; (2) immediately proximal to the elbow in the upper arm.	4 equidistant ROIs (size = 1.5 mm × 1.5 mm) along the imaged nerve	m/s
	Lin 2022 [62]	Aixplorer Supersonic Imagine	4–15 MHz and 40 mm linear transducer	L	Midpoint of the forearm	2 mm diameters ROI	kPa
	Lee 2019 [63]	Siemens Acuson S2000	9L4 Linear Array Transducer	T	Carpal tunnel inlet	2 × 2 mm ROI	m/s
	Staber 2022 [61]	Canon Aplio i800	14 MHz linear transducer	L	3 cm proximal to the flexor retinaculum	2 mm	m/s
	Rugel 2020 [37]	Aixplorer Supersonic Imagine	4 to 15-MHz linear array probe	L	Lower third of the biceps and forearm.	ROI of at least 1.5 cm in length and including the entirety of the nerve visible within 0.25 cm of the SW elastography box border.	m/s
***Ulnar nerve***							
	Durand 2021 [60]	Aixplorer Supersonic Imagine	5–18 MHz linear array transducer (SuperLineal SL18-5)	L	Immediately proximal to the medial epicondyle	ROI of 2 mm	kPa
	Wolny 2022 [57]	Aixplorer 12.2.0 Supersonic Imagine	Linear transducer array 2–10 MHz; SuperLinear 10-2	T	Ulnar tunnel	-	kPa
	Rugel 2020 [37]	Aixplorer Supersonic Imagine	4 to 15-MHz linear array probe	L	Lower third of the biceps and forearm.	ROI of at least 1.5 cm in length and including the entirety of the nerve visible within 0.25 cm of the SW elastography box border.	m/s
***Sciatic nerve***							
	Hirata 2020 [53]	Siemens Acuson S2000	Linear transducer array 9 L4 Transducer, 4–9 MHz	L	At 60% of the thigh length from the greater trochanter to the popliteal crease.	ROI as large as possible while excluding nontarget tissues.	m/s
	Neto 2019 [49]	Aixplorer 10.0 Supersonic Imagine	Linear array transducer SL 10-2 MHz, Super Linear 15-4	L	Posterior thigh, 10 cm below the gluteal fold.	The largest area within the epineurium boundaries in the elastographic window	m/s
	Neto 2017 [50]	Aixplorer 10.0 Supersonic Imagine	Linear array transducer SL 10-2 MHz, Super Linear	L	Posterior thigh, 10 cm below the gluteal fold.	The largest area within the epineurium boundaries in the elastographic window	m/s
	Andrade 2016 [59]	Aixplorer 6.1 Supersonic Imagine	L10-2 MHz, Super Linear transducer	L	7–10 cm distal to the gluteal fold	The largest area within the epineurium boundaries in the elastographic window	m/s
	Andrade 2022 [54]	Aixplorer 6.1 Supersonic Imagine	L10-2 MHz, Super Linear transducer	L	Landmarks: (i) midpoint between the ischial tuberosity and the greater trochanter; (ii) SN bifurcation. Points of measure: -Sciatic PROXIMAL and Sciatic DISTAL: obtained by dividing into 2 regions between (i) and (ii).	The largest area within the epineurium boundaries in the elastographic window	m/s
	Andrade 2018 [58]	Aixplorer 6.1 Supersonic Imagine	L10-2 MHz, Super Linear transducer	L	Proximal third of the thigh	The largest nerve area	m/s
***Tibial nerve***							
	Kawanishi 2022 [52]	Canon Aplio 300	10-MHz linear probe (PLT-1005BT)	L	1 cm superior to the medial malleolus	3 randomly selected ROI	m/s
	Anegawa 2023 [56]	Canon Aplio 300	10-MHz linear transducer (PLT-1005BT)	L	1 cm superior to the medial malleolus	3 randomly selected ROI	m/s
	Greening 2017 [36]	Siemens Acuson S2000	4–9 MHz 38.5 mm linear array transducer	L	TN: immediately proximal to the tarsal tunnel	4 equidistant ROIs (size = 1.5 mm × 1.5 mm) along the imaged nerve	m/s
	Andrade 2022 [54]	Aixplorer 6.1 Supersonic Imagine	L10-2 MHz, Super Linear transducer	L	Landmarks: (ii) SN bifurcation; (iii) lateral femoral condyle; and (iv) the medial malleolus. Points of measure: -Tibial PROXIMAL: between (ii) and (iii) -Tibial INTERMEDIATE, and Tibial DISTAL: at 50% and 10% of the distance from (iv) and (iii).	The largest area within the epineurium boundaries in the elastographic window	m/s

L = longitudinal; T = transverse; ROI = region of interest; SN = sciatic nerve; TN = tibial nerve.

**Table 4:** Median nerve stiffness in response to joint movement.

*Median Nerve*					
Author and Year	N	Location	Initial Position	Movement and Involved Joints	Results
Zhu 2018 [55]	40	Forearm	Seated with the arm extended. Elbow flexed 90°, the forearm in supine position, and wrists relaxed on a flat surface with fingers semi- flexed (Posture 1)	Wrist stretched maximally (extension) while maintaining the forearm on the flat surface (Posture 2).	Significant effect of the different nerve postures was observed, and the MN in the tension condition had a higher stiffness than that in the slack condition (*p* < 0.001).
Greening 2017 [36]	26 (n = 18 for position 2 of MN evaluation)	Forearm Proximal Elbow	Supine, with the shoulder abducted to 30°, elbow flexed to 90°, and the wrist in maximum flexion (50–60°) (Position 1)	(Position 2) shoulder abduction to 90° while maintaining 90° elbow flexion and maximum wrist flexion (not included in the article data analysis); (Position 3) the wrist was extended to end of range (60–70°), while maintaining 90° shoulder abduction and 90° elbow flexion; (Position 4) the elbow was extended maximally (135–190°) while maintaining 90° shoulder abduction and maximum wrist extension	Position 1: the mean SWV was 2.22 ± 0.07 m/s in the upper arm and 2.61 ± 0.08 m/s in the forearm, a difference that was significant. Position 2: negligible change in the MN SWV in the upper arm (mean = 2.59 ± 0.11 m/s), whereas in the forearm, there was a small but significant decrease. Position 3: increase in MN SWV in the upper arm (mean = 3.10 ± m/s) and forearm (mean = 5.87 ± 0.19 m/s). In this position, the percent increase from position 1 was significantly higher in the forearm (127 ± 7%) compared to the upper arm (40 ± 4%). Position 4: substantial increase in MN SWV in both the upper arm (mean = 6.80 ± 0.31 m/s; percent increase from position 1 = 208 ± 13%) and forearm (mean = 8.65 ± 0.19 m/s; percent increase from position 1 = 236 ± 10%). Elbow angle did not correlate with MN SWV in the forearm or upper arm.
Lin 2022 [62]	20	Forearm	On a chair with their upper arms positioned horizontally, with the shoulder abducted 90 degrees and 90 degrees externally rotated.	The MN was imaged during elbow extension in the following postures: (Position 1) with neutral posture, (Position 2) with wrist extension, (Position 3) with contralateral cervical flexion, and (Position 4) with both wrist extension and contralateral cervical flexion.	The mean shear modulus of the MN in the middle forearm was 137.71 ± 22.72 kPa at the neutral posture, only contralateral cervical flexion was 211.00 ± 30.49 kPa, and only wrist extension was 252.34 ± 40.30 kPa and 297.35 ± 64.60 kPa at contralateral cervical flexion + wrist extension.
Lee 2019 [63]	26	Wrist	Elbow at 90°	Six finger/wrist combinations: (A) wrist neutral (0°), finger neutral; (B) wrist neutral, finger grasp; (C) wrist neutral, finger extension; (D) wrist extension (30°), finger neutral; (E) wrist extension, finger grasp; and (F) wrist extension, finger extension.	Significant differences in SWV in all six motions (*p* < 0.001) showing an increasing trend from (A) to (F): (A) 2.3 ± 0.5 m/s; (B) 2.7 ± 0.5 m/s; (C) 2.7 ± 0.4 m/s; (D) 2.9 ± 0.5 m/s; (E) 3.0 ± 0.5 m/s; (F) 3.1 ± 0.5 m/s.
Staber 2022 [61]	11	Forearm	Seated with the back of the hand on the cushion and elbow flexed at 120°.	Three positions in the wrist joint: neutral (0°), individual maximal flexion and maximal extension.	SWV was higher in extension (5.8 m/s) than in flexion (3.2 m/s) as well as in neutral position (3.8 m/s) (extension vs. flexion (*p* < 0.001), extension vs. neutral (*p* < 0.002) and neutral vs. flexion (*p* = 0.071).
Rugel 2020 [37]	16	Proximal Elbow Forearm	Supine on an examination table with their shoulder at 45° abduction and wrist at neutral position.	Extension and 90° elbow flexion.	89.3% increase in MN SWV in the proximal elbow and 64.0% increase in the forearm with elbow extension compared to flexion (*p* < 0.01).

MN = median nerve; SWV = shear-wave velocity.

**Table 5:** Summary table of sensitivity analysis performed by removing one study at a time in meta-analyses with n ≥ 3 studies.

***Median Nerve—Wrist Extension vs. Neutral: SMD [95%CI]: 3.16 [1.20, 5.12], I^2^: 95%***			
**Study Removed**	**SMD (95%CI) without the Study**	**I^2^ without the Study**	**% Variation of Effect Size**
Lee 2019 [63]	3.71 [1.25, 6.18]	94%	17.41%
Lin 2022 [62]	3.08 [0.43, 5.73]	96%	2.53%
Staber 2022 [61]	3.66 [1.05, 6.27]	96%	15.82%
Zhu 2018 [55]	2.19 [1.07, 3.31]	80%	30.70%
***Ulnar Nerve—Elbow Extension vs. Elbow 90° Flexion: SMD [95%CI]: 2.91 [1.88, 3.95], I^2^: 70%***			
**Study Removed**	**SMD [95%CI] without the Study**	**I^2^ without the Study**	**% Variation of Effect Size**
Durand 2021 [60]	2.96 [1.37, 4.55]	85%	1.72%
Rugel 2020 [37]	3.39 [2.46, 4.31]	38%	16.49%
Wolny 2022 [57]	2.37 [1.64, 3.09]	0%	18.56%
***Sciatic Nerve—Ankle Dorsiflexion vs. Plantar Flexion: SMD [95%CI]: 1.08 [0.72, 1.44], I^2^: 22%***			
**Study Removed**	**SMD [95%CI] without the Study**	**I^2^ without the Study**	**% Variation of Effect Size**
Andrade 2016 [59]	0.98 [0.67, 1.29]	0%	9.26%
Andrade 2018 [58]	1.17 [0.72, 1.63]	36%	8.33%
Hirata 2020 [53]	1.26 [0.86, 1.65]	0%	16.67%
Neto 2017 [50]	0.97 [0.56, 1.39]	17%	10.19%
Neto 2019 [49]	1.08 [0.65, 1.51]	40%	0%
***Sciatic Nerve—Ankle Dorsiflexion vs. Neutral (≈0°): SMD [95%CI]: 0.56 [0.26, 0.85], I^2^: 0%***			
**Study Removed**	**SMD [95%CI] without the Study**	**I^2^ without the Study**	**% Variation of Effect Size**
Andrade 2018 [58]	0.58 [0.23, 0.93]	8%	3.57%
Hirata 2020 [53]	0.73 [0.32, 1.13]	0%	30.36%
Neto 2017 [50]	0.46 [0.10, 0.81]	0%	17.86%
Neto 2019 [49]	0.52 [0.21, 0.83]	0%	7.14%
***Sciatic Nerve—Ankle Neutral (≈0°) vs. Plantar Flexion: SMD [95%CI]: 0.44 [0.15, 0.74], I^2^: 0%***			
**Study Removed**	**SMD [95%CI] without the Study**	**I^2^ without the Study**	**% Variation of Effect Size**
Andrade 2018 [58]	0.47 [0.15, 0.79]	0%	6.82%
Hirata 2020 [53]	0.48 [0.09, 0.88]	0%	9.09%
Neto 2017 [50]	0.36 [0.01, 0.71]	0%	18.18%
Neto 2019 [49]	0.46 [0.15, 0.77]	0%	4.55%

**Table 6:** Ulnar nerve stiffness in response to joint movement.

*Ulnar Nerve*					
Author and Year	N	Location	Initial Position	Movement and Involved Joints	Results
Rugel 2020 [37]	16	Proximal Elbow Forearm	Supine on an examination table with their shoulder at 45° abduction and wrist at neutral position.	Extension and 90° elbow flexion.	91.1% increase in UN SWV in the proximal elbow and 37.4% increase in the forearm with elbow flexion compared to extension (*p* < 0.01).
Durand 2021 [60]	11	Proximal Elbow	-	0°, 45°, 90° and 120° elbow flexion.	Significant differences in the shear elastic modulus between 0° (mean 37.1 ± 9.2 kPa) and 45° of elbow flexion (mean 70.3 ± 32.1 kPa, *p* < 0.01), between 45° and 90° (mean 100.3 ± 29.4 kPa, *p* < 0.05), and between 90° and 120° (mean 141.7 ± 31.7 kPa, *p* < 0.005).
Wolny 2022 [57]	31	Elbow	Side lying	Full extension, 45°, 90° and maximal elbow flexion.	Share modulus increases with increasing degrees of elbow joint flexion.

UN = ulnar nerve; SWV = shear-wave velocity.

**Table 7:** Sciatic nerve stiffness in response to joint movement.

*Sciatic Nerve*					
Author and Year	N	Location	Initial Position	Movement and Involved Joints	Results
Hirata 2020 [53]	20 young males and 20 older males	Proximal thigh	Hips and knees extended	3 different ankle positions: (1) 30° ankle plantar flexion; (2) neutral; and (3) 15° dorsal flexion.	SWV values were lower in older than in young participants at any joint angle (*p* ≤ 0.024, d ≥ 0.748). For both groups, SWV values became higher as the ankle dorsiflexed. SWS values at the maximal dorsiflexion angle were lower in older participants than in young participants. *Young males mean SWV (m/s)*: (1) 2.66 ± 0.61; (2) 3.09 ± 1.12; (3) 3.77 ± 1.71. *Older males mean SWV (m/s)*: (1) 1.92 ± 0.29; (2) 2.12 ± 0.33; (3) 2.47 ± 0.37.
Neto 2019 [49]	8 patients with chronic unilateral low back-related leg pain 8 healthy controls	Proximal thigh	Prone position	9 ankle position between 0% and 80% of maximum ankle dorsiflexion (0% = 40° ankle plantar flexion)	SN stiffness of the affected limb of patients with chronic low back-related leg pain is higher than that of the unaffected limb. No differences were observed between the unaffected limb of people with low back-related leg pain and the healthy controls. *Control group left side SWV (m/s)*: 0% 6.93 ± 1.65; 10% 6.89 ± 1.61; 20% 6.91 ± 1.64; 30% 7.06 ± 1.42; 40% 7.26 ± 1.30; 50% 7.43 ± 1.19; 60% 7.98 ± 1.06; 70% 8.41 ± 1.16; 80% 8.67 ± 1.28. *Control group right side SWV (m/s)*: 0% 7.02 ± 1.63; 10% 6.82 ± 1.50; 20% 6.93 ± 1.43; 30% 6.91 ± 1.51; 40% 7.38 ± 1.65; 50% 7.92 ± 1.69; 60% 8.34 ± 1.54; 70% 9.00 ± 1.74; 80% 9.11 ± 1.70. *Patients group affected side SWV (m/s)*: 0% 7.51 ± 1.73; 10% 7.62 ± 1.96; 20% 7.81 ± 2.07; 30% 7.82 ± 2.00; 40% 8.11 ± 2.07; 50% 8.49 ± 2.32; 60% 9.22 ± 2.30; 70% 10.03 ± 2.12; 80%10.91 ± 2.92. *Patients group unaffected side SWV (m/s)*: 0% 6.85 ± 1.81; 10% 6.89 ± 1.81; 20% 6.79 ± 1.54; 30% 7.12 ± 1.64; 40% 7.27 ± 1.88; 50% 7.46 ± 1.92; 60% 7.77 ± 2.29; 70% 8.77 ± 1.97; 80% 9.73 ± 2.37.
Neto 2017 [50]	14	Proximal thigh	Prone position	9 ankle position between 0% and 80% of maximum ankle dorsiflexion (0% = 40° ankle plantar flexion)	Increased SWV at 50 to 80% of ankle ROM compared to the 0% of ankle ROM in both the experimental (*p* = 0.04) and control (*p* = 0.01) limbs in the pre intervention (*p* = 0.01).
Andrade 2016 [59]	10	Proximal thigh	2 knee positions: Knee in full extension and knee in 90° flexion.	Ankle passively moved from 40° of plantarflexion to 80% of the maximal range of dorsiflexion (80% max. dorsiflexion = 100%).	The SWV of the SN significantly increased (*p* < 0.0001) during dorsiflexion when the knee was extended, but no changes were observed when the knee was flexed to 90°. For knee extension, the SWV was significantly higher at 70%, 80%, 90%, and 100% of ankle angle relative to 0% (*p* ≤ 0.002). SWV was significantly greater for knee 180° vs. knee 90° across all ankle angle increments (every 10% from 0% to 100%) (*p* values ranging from 0.001 to 0.002).
Andrade 2022 [54]	60	Proximal thigh Mid-thigh	(1) Hip neutral in supine position, knee and ankle in full-extension and neutral position	(2) Hip flexed at 90° with knee and ankle in full-extension and neutral position.	SWV increased with hip flexion (average increase +54.3%; *p* < 0.0001), but the increase was not different among nerve locations (*p* = 0.233). SWV increase of +2.4 ± 1.6 m/s for SN at the proximal thigh and +2.8 ± 1.9 m/s at the mid-thigh. *Proximal thigh SWV (m/s)*: 4.6 ± 1.2 (hip neutral), 7.1 ± 1.2 (hip flexed). *Mid-thigh SWV (m/s)*: 5.7 ± 1.2 (hip neutral), 8.5 ± 1.6 (hip flexed).
Andrade 2018 [58]	15	Proximal thigh	Supine with the hip in neutral position.	Progressive ankle dorsiflexion, SN imaged every 2°, from 40° of plantar flexion to the maximal ankle ROM in dorsiflexion	Exponential increase in SN stiffness during passive ankle dorsiflexion while the participants were positioned in hip-neutral position.

SWV = shear-wave velocity; SN = sciatic nerve; ROM = range of motion.

**Table 8:** Tibial nerve stiffness in response to joint movement.

*Tibial Nerve*					
Author and Year	N	Location	Initial Position	Movement and Involved Joints	Results
Kawanishi 2022 [52]	20	Distal leg	Intermediate position of the trunk and neck, 90° hip flexion, 30° knee flexion.	5 ankle positions: maximum dorsiflexion (100% DF), plantar flexion in the resting position (0% DF), and 3 points (25% DF, 50% DF, and 75% DF), which divided the range of motion from 0% DF to 100% DF.	SWV increased with dorsiflexion. Significant differences in SWV between 0% and 75% DF, 0% and 100% DF, and 25% and 100% DF. Significant negative correlation between the maximum ankle dorsiflexion and stiffness of the TN at 100% DF (*p* = 0.01) and 75% DF (*p* = 0.002). The SWV at 75% DF and 100% DF was higher in participants with lower maximal ankle dorsiflexion. Significant negative correlation between the total ankle range of motion and stiffness of the nerve at each joint angle. The SWV at each joint angle was high among the study participants with a reduced total ankle range. SWV (m/s): 0% DF: 4.5 ± 1.7 m/s; 25% DF: 5.2 ± 1.6 m/s; 50% DF: 6.2 ± 1.5 m/s; 75% DF: 7.0 ± 1.1 m/s; and 100% DF: 7.5 ± 0.7 m/s.
Anegawa 2023 [56]	21	Distal leg	Hip at 90° with 30° of knee flexion.	Two ankle positions: 25% and 75% of the dorsiflexion angle (25% DF and 75% DF).	The 75% DF-SWV was significantly greater than the 25% DF-SWV (7.4 ± 0.7 m/s versus 5.5 ± 1.3 m/s, *p* < 0.001).
Andrade 2022 [54]	60	Popliteal fossa Proximal leg Distal leg	(1) Hip neutral in supine position, knee and ankle in full-extension and neutral positions.	(2) Hip flexed at 90° with knee and ankle in full-extension and neutral positions.	Average increase in SWV by 54.3% (*p* < 0.0001). The increase was not different among nerve locations (*p* = 0.233). SWV increase of +2.4 ± 1.6 m/s for SN at the popliteal fossa 2.5 ± 1.9 m/s at the proximal leg and + 2.9 ± 1.8 m/s at the distal leg. *Popliteal fossa SWV (m/s)*: 6.5 ± 1.5 (hip neutral); 9.4 ± 2.0 (hip flexed). *Proximal leg SWV (m/s)*: 5.8 ± 1.1 (hip neutral); 8.2 ± 1.7 (hip flexed). *Distal leg SWV (m/s)*: 6.2 ± 1.0 (hip neutral); 9.1 ± 1.5 (hip flexed).
Greening 2017 [36]	26	Distal leg	(1) Hip neutral, knee flexed to 90°, and foot neutral.	(2) knee maximally extended, and the ankle was dorsiflexed to end of range, while maintaining the hip in neutral; (3) the hip was positioned into maximum flexion (40–90°) while maintaining maximum knee extension and ankle dorsiflexion.	(Position 1) mean SWV: 3.25 ± 0.10 m/s. (Position 2) caused a significant increase by 60 ± 6% in SWV (mean = 5.16 ± 0.21 m/s). (Position 3) further increase (136 ± 9% from starting position) in TN SWV (mean = 7.57 ± 0.28 m/s). Hip angle did not correlate with TN SWV (rs = 0.22).

TN = tibial nerve; SWV = shear-wave velocity.

## Supplementary Materials

The following supporting information can be downloaded at: https://www.mdpi.com/article/10.3390/diagnostics14030343/s1, File S1: PRISMA 2020 Checklist. File S2: The search strategy.
