# Supplementary material for: In Vivo Effects of Joint Movement on Nerve Mechanical Properties Assessed with Shear-Wave Elastography: A Systematic Review and Meta-Analysis
Source: Diagnostics (Basel). 2024 Feb 5;14(3):343. doi: 10.3390/diagnostics14030343 (PMC10855485; doi:10.3390/diagnostics14030343)
Supplement: Supplementary file 1 [file diagnostics-14-00343-s001.zip › File S2.pdf]

S2 – Search strategy

Search date: 10/07/2023

- **Pubmed**

("Shearwav*" OR "Shear Wav*" OR "Shear-Wav*" OR "Shear wav*" OR "Sonoelastograph*" OR "Acoustic radiation force impulse" OR "ARFI" OR "elastograph*" OR "shear imaging" OR "Elasticity Imaging Techniques"[Mesh]) AND ("Nerv*" OR "neural tissue" OR "nervous system" OR "Neurodynami*" OR "Peripheral Nervous System"[Mesh]) AND ("position*" OR "postur*" OR "movement" OR "Angle" OR "Motion" OR "Tension" OR "stretch*" OR "excursion" OR "biomechanic*" OR "Posture"[Mesh] OR "Movement"[Mesh] OR "Motion"[Mesh])

- **EMBASE**

('shearwav*':ti,ab,kw OR 'shear wav*':ti,ab,kw OR 'shear-wave*':ti,ab,kw OR 'sonoelastograph*':ti,ab,kw OR 'acoustic radiation force impulse':ti,ab,kw OR 'arfi':ti,ab,kw OR 'elastograph*':ti,ab,kw OR 'shear imaging':ti,ab,kw OR 'elastograph'/exp) AND ('nerv*':ti,ab,kw OR 'neural tissue':ti,ab,kw OR 'nervous system':ti,ab,kw OR 'neurodynami*':ti,ab,kw OR 'nerve'/exp OR 'peripheral nervous system'/exp) AND ('position*':ti,ab,kw OR 'postur*':ti,ab,kw OR 'movement':ti,ab,kw OR 'angle':ti,ab,kw OR 'motion':ti,ab,kw OR 'tension':ti,ab,kw OR 'stretch*':ti,ab,kw OR 'excursion':ti,ab,kw OR 'biomechanic*':ti,ab,kw OR 'movement (physiology)'/exp OR 'body position'/exp)

- **Scopus**

TITLE-ABS-KEY (("Shearwav*" OR "Shear Wav*" OR "Shear-Wav*" OR "Sonoelastograph*" OR "Acoustic radiation force impulse" OR "ARFI" OR "elastograph*" OR “shear imaging”) AND ("Nerv*" OR "neural tissue" OR "nervous system" OR “Neurodynami*”) AND ("position*" OR "postur*" OR "movement" OR "Angle" OR "Motion" OR "Tension" OR "stretch*" OR “excursion” OR “biomechanic*”))

- **Web of Science**

TS=(("Shearwav*" OR "Shear Wav*" OR "Shear-Wav*" OR "Sonoelastograph*" OR "Acoustic radiation force impulse" OR "ARFI" OR "elastograph*" OR “shear imaging”) AND ("Nerv*" OR "neural tissue" OR "nervous system" OR “Neurodynami*”) AND ("position*" OR "postur*" OR "movement" OR "Angle" OR "Motion" OR "Tension" OR "stretch*" OR “excursion” OR “biomechanic*”))

- **Science direct**

("shear wave" OR "elastography") AND ("nerve" OR "neural tissue") AND ("position" OR "Motion" OR "movement" OR "angle" OR "posture")

- **Cochrane Library**

#1 (“shear wave” OR “shear waves” OR “shearwave” OR “shearwaves” OR “shear-wave” OR “shear-waves” OR “acoustic radiation force impulse” OR “ARFI” OR “shear imaging” OR sonoelastography OR elastography) 1048
#2 MeSH descriptor: [Elasticity Imaging Techniques] explode all trees 216
#3 #1 OR #2 1123
#4 ("Nerve" OR "nerves" OR "neural tissue" OR "nervous system" OR “Neurodynamic”) 73240
#5 MeSH descriptor: [Nervous System] explode all trees
#6 #4 OR #5
#7 ("position" OR “positions “OR "posture" OR “postures” OR "movement" OR "Angle" OR "Motion" OR "Tension" OR "stretch" OR “excursion” OR “biomechanics”)
#8 MeSH descriptor: [Movement] explode all trees
#9 MeSH descriptor: [Posture] explode all trees
#10 MeSH descriptor: [Range of Motion, Articular] explode all trees
#11 #7 OR #8 OR #9 OR #10
#12 #3 AND #6 AND #11
